# Food Selectivity in Children with Autism Spectrum Disorder and in Typically Developing Peers: Sensory Processing, Parental Practices, and Gastrointestinal Symptoms

**DOI:** 10.3390/nu17172798

**Published:** 2025-08-28

**Authors:** Paolo Mirizzi, Marco Esposito, Orlando Ricciardi, Domenico Bove, Roberta Fadda, Alessandro O. Caffò, Monica Mazza, Marco Valenti

**Affiliations:** 1 Department of Education, Psychology, Communication, University of Bari, 70121 Bari, Italy; paolomirizzi@yahoo.it (P.M.); alessandro.caffo@uniba.it (A.O.C.); 2 Cinetic Center, Neuromotor Rehabilitation Centre, Via Santella 26, 81025 Marcianise, Italy; 3 Autism Research and Treatment Centre, Una Breccia Nel Muro, 00168 Rome, Italy; orlando.ricciardi@unabreccianelmuro.org; 4 Department of Applied Clinical Sciences and Biotechnology, University of L’Aquila, 67100 L’Aquila, Italy; monica.mazza@univaq.it (M.M.); marco.valenti@univaq.it (M.V.); 5 FUSIS MCF (Clinic Neuroscience Research and Training), 81100 Caserta, Italy; fusismcf@gmail.com; 6 Department of Pedagogy, Psychology, Philosophy, University of Cagliari, 09100 Cagliari, Italy; robfadda@unica.it; 7 Regional Centre for Autism, Abruzzo Region Health System, 67100 L’Aquila, Italy

**Keywords:** food selectivity, autism spectrum disorder, sensory processing, diet, gastrointestinal disorders, BMI, children, parental practices

## Abstract

Background/Objectives: Food selectivity is a prevalent and challenging issue in childhood, particularly in children with autism spectrum disorder (ASD), which may result in restricted dietary patterns and nutrient deficiencies. This study aimed to identify high-risk subgroups of children by combining food selectivity, diet, BMI, gastrointestinal symptoms, sensory processing, and parental feeding practices in children with ASD and in typically developing children (TDC). Methods: To achieve this aim, we ran a cross-sectional, survey-based study, including 408 children (aged 3 to 12.11 years), with gender-matched groups. Both parents completed a survey on children’s diet, anthropometric curves, gastrointestinal symptoms, and the Brief Autism Mealtime Behavior Inventory (BAMBI), Short Sensory Profile (SSP), and Caregiver’s Feeding Style Questionnaire (CFSQ). Data analysis included comparative tests, correlations, and k-means cluster analysis. Results: Children with ASD exhibited significantly greater sensory processing difficulties, higher food refusal, limited food variety in the diet, and autism-related mealtime characteristics compared with TDC across all age groups. Caregivers of children with ASD reported higher controlling and contingency management feeding practices compared to the parents of the TDC. We found a strong correlation between sensory sensitivities and feeding issues. Notably, Body Mass Index (BMI) was not significantly associated with dietary restriction or gastrointestinal symptoms. Cluster analysis revealed a high-risk sub-phenotype in both groups of children with some differences, characterized by high food selectivity, taste, tactile, and smell sensitivity, gastrointestinal symptoms, and overactive parental practices. Conclusions: The early identification of this subgroup might foster more tailored, multidisciplinary, and effective assessment and clinical intervention.

## 1. Introduction

In early childhood, the establishment of eating behaviors is a complex developmental process, characterized by a heterogeneous repertoire that typically aligns with proper developmental milestones and nutritional rhythms. However, a significant proportion of young children present with irregular eating patterns, such as reduced appetite (inappetence), which can be challenging to interpret and manage accurately. Empirical data indicate that approximately one-quarter of children exhibiting typical psychomotor development will encounter some form of dietary issue during their early childhood years [1]. Of particular concern, children diagnosed with neurodevelopmental disorders are considered to be at a substantially elevated risk for the development of eating disorders when compared to typically developing children (TDC) [2,3].

### 1.1. Food Selectivity and Feeding Challenges in Children with Autism Spectrum Disorder

The most frequently observed dietary problems in children with autism spectrum disorder (ASD) include a delayed acquisition of autonomous feeding skills relative to developmental expectations, the manifestation of dysfunctional behaviors during mealtimes, and a restricted intake of food driven by pronounced preferences for specific food attributes. This latter phenomenon, widely recognized as food selectivity (also known as picky or choosy eating), involves consistently choosing foods with particular characteristics while rejecting those without those characteristics. The prevalence of feeding disorders in such a clinical population can be exceptionally high, reaching up to 89% [4]. Specifically, children with ASD often demonstrate strong aversions to foods based on a range of characteristics by type (specific brands or categories), texture (preference for crunchy or pureed consistencies), temperature, color, packaging, or cutlery. Food selectivity results in a significantly limited acceptance of foods and a more limited diet compared to TDC [5]. Moreover, the attempt to present them with non-preferred foods can elicit severe disruptive behavioral and emotional responses in children with ASD. These reactions may include emotional outbursts, challenging behaviors such as screaming, crying, irritability, self-directed aggression, aggression towards others, and attempts to escape the mealtime situation, distress reactions, or avoidance behaviors such as turning away, chewing without swallowing, spitting, or vomiting [6,7,8,9,10,11]. The acute stress associated with these mealtime difficulties, both in the children and in their caregivers, can profoundly inhibit the successful introduction of novel foods, thereby diminishing the likelihood of achieving a varied diet over time [12,13,14]. Mothers of children with ASD often describe the mealtime routine as a significant source of stress, and lacking positive interpersonal interactions, along with the need to reduce the food variability for the entire family [15].

### 1.2. Food Selectivity and Anthropometric Growth Trajectories and Gastrointestinal Issues

The relationship between food selectivity and anthropometric measures has been extensively investigated. However, the results are still controversial. Some studies identified a risk of both under- and over-nutrition in children with food selectivity. A case–control study [16] evaluated 55 children with ASD and 91 TDC aged between 6 and 18 years, finding food selectivity in 60.6% of the ASD group versus 37.9% of controls; among children with ASD, 18.4% were underweight and 16.3% were obese, compared to 5.5% and 6.6% in the control group, respectively, while nutrient analyses revealed deficits in fiber, iron, calcium, and vitamins A, D, and E. Similarly, in a cross-sectional study of 90 children with ASD aged 2–10 [17], the authors reported food refusal in 57.8% of the sample. They found that 40% were overweight or obese, with problematic mealtime behaviors significantly associated with elevated Body Mass Index (BMI). In contrast, Sharp and colleagues [18], studying 70 children aged 2–17 years with ASD and severe food selectivity found that despite extreme dietary limitations (e.g., 27% consumed no fruits; 67% consumed no vegetables), the majority exhibited normal growth trajectories, suggesting that energy intake may remain adequate despite poor diet quality. In particular, another research group, examining 53 children with ASD and 58 TDC aged 3–11, reported higher intake of sweetened beverages and snacks and lower fruit/vegetable consumption in the ASD group, yet observed only weak associations between these dietary differences and BMI scores [19]. A broader perspective is offered [20], where researchers analyzed data from a large sample of children in the Generation R cohort. While higher autistic traits were associated with food-avoidant behaviors (e.g., picky eating, slow eating), they were not predictive of increased BMI. On the other hand, research has consistently demonstrated that food selectivity in ASD is associated with inadequate intake of essential nutrients, including protein, calcium, vitamins B12 and D, fiber, and minerals such as iron and selenium where selective eaters with ASD were more likely to be at risk for at least one severe nutrient deficiency [21]. A meta-analysis [22] reinforced these findings by highlighting consistent patterns of lower protein, calcium, phosphorus, vitamin D, and B vitamin consumption among children with ASD across multiple studies. Given the critical role of balanced nutrition in physical and cognitive development, a thorough understanding of how food selectivity contributes to nutritional deficiencies is essential for designing effective dietary interventions tailored to the needs of children with ASD.

In addition, recent research has highlighted the significant comorbidity with gastrointestinal symptoms (GIS), where a secondary effect of food selectivity is the alteration of the gut–brain axis in children with ASD. More precisely, autistic behaviors related to selectivity interact with the gut microbiome and the occurrence of gastrointestinal symptoms in a vicious circle, which has recently gained focus on the link between gastrointestinal symptoms, autism, and microbiota [23]. The authors describe a feedback system between the composition of the gut microbiota, altered by food selectivity patterns, and its potential effects on ASD symptoms through the gut–brain axis. Another study supports the possibility that autistic traits contribute to gastrointestinal symptoms. A prospective cohort study that followed participants from fetal life to young adulthood in a multi-ethnic urban population supported a link between the presence of autistic traits at age six and the development of constipation symptoms at age 10 [24]. In addition, the analyses performed suggested that food selectivity explained the predictive role of autistic traits in constipation symptoms. Indeed, this result confirms a vicious circle between autistic traits, food selectivity, and gastrointestinal symptoms. Furthermore, the relationship between autism and GIS has been previously found in other studies, including a population-based analysis comparing three groups of subjects: ASD, developmental delay (DD), and controls. The results of the study showed that children with ASD were more likely to have experienced gastrointestinal symptoms as infants and toddlers than TDC and more likely to have had constipation than children with DD [25]. In summary, these findings suggest that food selectivity in children with ASD is a pervasive feature, which might lead to both optimal or at-risk dietary habits, nutritional outcomes, and growth trajectories, thanks to a complex combination with neurodevelopmental symptoms, emotional regulation, and broader environmental factors.

### 1.3. Food Selectivity and Sensory Processing in Children with ASD

In autistic children, food selectivity is associated with a constellation of co-occurring features, including sensory over-responsivity, behavioral rigidity, and GIS that significantly contribute to or exacerbate selective eating behaviors [26]. Sensory over-responsivity (or sensory hypersensitivity) refers to heightened and often aversive responses to sensory stimuli as taste and texture (e.g., mushy, crunchy, or slimy), intense tastes (e.g., bitter or spicy), or strong odors from certain foods may provoke discomfort or nausea, and the appearance of foods or the sounds during mealtimes (e.g., chewing, utensil clinking) can also be distressing [27]. These sensory challenges can lead to a highly restricted diet, often limited to foods with predictable textures and flavors (e.g., dry, bland, or processed foods) [28]. Behavioral rigidity is characterized by a strong preference for sameness, routines, and predictability, which are core characteristics of autism. This preference is often evident in individuals with ASD, such as a strong preference for specific brands, shapes, or colors of food (e.g., a specific type of pasta or a particular brand of crackers) [29]. In addition to resistance to trying new foods (food neophobia), even minor changes in food presentation can lead to refusal [30]. Changes in seating, dishware, or meal timing can also trigger distress and non-compliance. This rigidity makes it difficult to introduce variety into the child’s diet, maintaining or reinforcing selective eating patterns. Concerning gastrointestinal issues, pain or discomfort during or after eating may lead the child to associate eating with negative experiences. Likewise, a limited dietary intake can exacerbate GIS, creating a cyclical pattern of distress and further restriction [31]. In summary, the relationship between sensory processing and food selectivity might be exacerbated by GIS. Sensory issues may lead to avoiding a variety of foods, such as fiber-rich foods. Dietary restrictions might contribute to GIS, for example, constipation. GIS may increase anxiety and feelings of discomfort or even sickness after eating, which in turn might foster even higher food selectivity. Breaking this dysfunctional circle is crucial for effective intervention [32].

### 1.4. Food Selectivity and Parental Feeding Style in Children with ASD

Caregiver feeding styles influence the child’s eating behaviors and the mealtime environment. Caregivers frequently adopt authoritarian or emotionally charged feeding approaches, including pressure to eat and use of food as a reward, often motivated by concern for their child’s nutritional sufficiency and adherence to expected mealtime behaviors [33]. However, these strategies may inadvertently reinforce food refusal and heightened food neophobia in children [34]. A systematic review considering the possible interventions to implement caregiver feeding style indicated that parent-led behavioral strategies can improve food acceptance and dietary variety in children. However, the heterogeneity of protocols and the lack of standardized measures of the nutritional outcome limit definitive conclusions regarding long-term health outcomes [35]. Moreover, atypical sensory processing in ASD, especially atypical oral sensory sensitivity, has been robustly associated with increased food avoidance and selective eating [36]. In the literature, a strong relationship exists between children’s eating habits and those of their parents, which can influence the eating style of the entire household. The authors conducted specific analyses to determine whether children’s eating behavior was related to the severity of diagnostic symptoms of autism or whether it was related to the family’s food preferences. The results of these analyses showed that family food preferences were the only significant predictor of children’s food preferences, suggesting that families who followed more restrictive diets had children with more restrictive eating behaviors [15]. In summary, the findings of these studies highlight the complexity of feeding challenges in ASD, which seems to be still not adequately addressed. More research is needed to understand the complex combined effect of the specific sensory needs of this population and the different parental feeding styles.

### 1.5. Aims of the Study and Research Questions

As it is possible to see from the studies analyzed so far, several questions are still open, along with critical methodological issues. Firstly, the definition of the phenomenon is still not straightforward. The clinical definition is comprehensive, since it encompasses food refusal, consuming only a single food, and a restricted variety or type of food. Moreover, very often different terms such as food selectivity, picky eating, and fussy eating have been used interchangeably [37]. In addition, previous studies applied a variety of measures, including indirect (i.e., survey, interview, questionnaires, checklist, and diaries) and direct measurements (i.e., trial-based). The variety of measures applied so far makes it difficult to evaluate the convergent validity of the results of previous studies in identifying the food selectivity [38], requiring the researchers to find more consistent ways to collect the data and more cooperation between different research groups [39]. Furthermore, previous studies revealed a gender bias and discrepancies between the reports collected from mothers, fathers, caregivers, and other tutors of children with ASD. Indeed, it seems that males tend to tolerate more negative behaviors than females, while mothers seem to perceive more deviant behaviors in their children than fathers [40]. Thus, researchers should consider multiple sources of information when using indirect measures. For this article, we ran a cross-sectional, survey-based, between-subject study comparing a group of children with ASD with a group of TDC, aiming to answer the following research questions, which are still open in the literature:

1. Do children with ASD show more food selectivity than TDC? Are there any differences between mothers and fathers in their children’s reports regarding the dimensions interviewed by the survey?
2. Is there an association between age, sex, sensory processing, BMI, GIS, parental feeding practices, and food selectivity? Which dimensions influence more than others do?
3. Is it possible to identify a higher-risk autism phenotype, which results from the combination of food selectivity, BMI, GIS, sensory anomalies, and parental practices?

## 2. Methods

The study followed a cross-sectional, survey-based, between-subjects design comparing children with ASD and TDC. The first researcher (certified behavior analyst) identified key subgroups within a population in his community (e.g., by age, gender, and diagnosis) and set a quota for the number of participants needed from each subgroup in a non-random method. Recruitment was carried out in collaboration with schools, rehabilitation centers, and autism associations in southern Italy. Participation was voluntary, and both parents of each child were invited to complete the study questionnaires in person, during scheduled sessions with trained research staff. The survey package included demographic and clinical information, dietary habits, gastrointestinal symptoms, and three standardized instruments.

### 2.1. Participants

At the beginning of the questionnaire, the parents had to declare whether the child had a clinical diagnosis of ASD according to the DSM-5, reporting symptom severity ranging from 1 to 3 [41]. In addition, the parents sent the diagnosis by email to the researchers, along with the consent form. Before selecting the defined sample, we decided to exclude children with ASD with a comorbidity of learning disorders, neurological or genetic syndromes, or feeding disorders. A total sample of 816 records, comprising 408 children, was analyzed, of which 33% of children had ASD and 67% were TDC. Both groups were aged between 3 and 12.11 years old. Previously, to compare groups, we paired the subsamples based on proportions, age, and sex; consequently, we reduced and classified all records into age classes, including preschool, primary, and middle school.

### 2.2. Ethical Aspects

The study adhered to the Declaration of Helsinki and received approval from the University of Bari Ethics Committee (Protocol UNIBA-10162018; 16 October 2018), following the ethical code of the Italian Psychology Association, Developmental and Educational Psychology session. All participants’ parents provided written informed consent before data collection, and confidentiality was strictly maintained. Participants could leave the study at any time prior to completion of the survey. No external funding was received, and the authors declare no conflicts of interest.

### 2.3. Procedure and Tools

After consent, the first researcher provided an adapted survey to all parents [42]. The items asked the following child’s information: age, gender, current clinical interventions (including their dosage in hours per month), weight/height, food categories assumed (such as carbohydrates, vegetables/legumes, fruit, dairy, and meat/fish), allergies, and intolerances. Furthermore, items regarding vomiting, diarrhea, constipation, and reflux (4-point scale: never, rarely, sometimes, and often), while for the other abovementioned items, caregivers indicated merely the presence of behavior (yes/no). Consequently, the parents responded to three standardized questionnaires, such as the Brief Autism Mealtime Behavior Inventory (BAMBI), as a measure of mealtime behaviors in children with ASD aged between 3 and 11 years [43]. The tool comprises 18 items that assess the frequency of the child’s eating behavior over the last six months on a 5-point Likert scale, ranging from 1 (never) to 5 (almost always), with three dimensions: Food Refusal, including escape behaviors emitted to avoid unpleasant food; Limited Variety, which identifies children’s reluctance to try new foods or foods prepared differently due to the texture and type; and Autism Characteristics, which evaluates the most typical behaviors of the autistic spectrum. Higher scores represent a greater frequency of problematic behaviors. The questionnaire has received some reviews that highlighted its internal and convergent validity with a variable factor structure [44]. As a result, we measured the three dimensions: Food Refusal included five items (my child cries or screams during meals/turns his face or body away from his food/spits out food/throws utensils and food/closes mouth tightly when food is presented); Limited Variety included eight items (my child is open to trying new foods/dislikes certain foods/prefers the same foods/prefers crunchy foods/prefers a variety of foods/wants foods to be served in a particular way/only likes sweet foods/prefers foods prepared in a certain way); and Autism Characteristics included five items (my child sits at the table until the end of the meal/is aggressive during meals/shows self-injurious behaviors/flexible about eating habits/refuses to eat foods that have to be chewed). A principal components analysis was performed with three established theoretical dimensions, which explained, respectively, 0.41%, 0.37%, and 0.22% of variance (root mean square of the residual, 0.08; chi-square, 514.88; *p* < α). Correspondingly, we detected a very low saturation between items of the Autism Characteristic scale and the third factor extracted, along with an inconsistent Cronbach’s alpha (0.78, 0.77, and 0.47). As a result, we removed the third scale of BAMBI for the following analysis.

Furthermore, we selected the Short Sensory Profile (SSP) to assess the hyper- or hypo-sensation of both groups [45]. This instrument includes 38 items and seven subscales: Tactile Sensitivity, Taste/Smell Sensitivity, Movement Sensitivity, Under-responsiveness/Seek Sensation, Auditory Filtering, Low Energy/Weakness, and Visual/Auditory Sensitivity. The questions range on a 5-point Likert scale (from 1 = never to 5 = always). The subscale score can be classified as Typical if it falls within 1 standard deviation (SD) of the reference mean, Probable Difference if it lies between 1 and 2 SD, and Definite Difference if it is 2 SD or more below the mean. Lower scores indicate greater sensitivity than those of controls. Recent psychometric studies do not encourage the use of the total score [46] and its current factor structure [47,48,49,50,51,52]. As a result, an alpha of Cronbach was performed for each scale: Tactile (0.68), Taste (0.82), Movement (0.63), Seek Sensation (0.76), Auditory Filtering (0.65), Weak (0.92), and Visual/Auditory Sensation (0.60), hence we considered all the dimensions as input variables.

Lastly, we selected the Caregiver’s Feeding Style Questionnaire (CFSQ) [53] for our educational styles analysis, as it has been shown to correlate with the diet [54] and feeding difficulties of children with ASD [55]. The instrument offers 19 items on a 5-point Likert scale (from 1 = never to 5 = always) and three subscales regarding some behaviors adopted by caregivers with their child during mealtimes. High Control (putting the child in the chair or begging the child to eat dinner), Contingency Management (promise preferred items or remove something away from the child other than food), and Child Centered (helps the child cutting the food, telling something positive about the food, and arranging the food and cutlery in tolerable way). A principal components analysis was performed with three established theoretical dimensions, which explained, respectively, 0.38, 0.32, and 0.30% of variance (root mean square of the residual, 0.06; chi-square, 325.86; *p* < α). Similarly, we observed a strong correlation between items and related factors. Likewise, the Cronbach’s alpha revealed similar results (0.75, 0.84, and 0.80); hence, we considered the three dimensions as input variables.

### 2.4. Data Analysis

Firstly, we stratified the sample via sex and age classes, matching each child with ASD with one of the TDC group via power analysis, and pairing the proportions of the two subsamples. Records with missing or inconsistent demographic or clinical information were excluded from analyses. The survey included dichotomous variables (yes/no) such as current interventions (cognitive, behavioral, speech therapy, psychomotricity, and psychoeducational), monthly hours of each treatment, food categories (carbohydrates, vegetables/legumes, fruit, dairy, and meat/fish), allergies, intolerances, and four GIS variables (ordinal). Furthermore, we calculated the BMI as the ratio of weight (kg) to squared height (m^2^), and BMI-z = ((BMI/M)^L−1^)/(L × S) since it varies with age and sex. The last value expresses each child’s relative position within the normative distribution by indicating the number of standard deviations the observed BMI deviates from the median of peers of the same age and sex [56]; we calculated a chi-squared test for contingency tables, adjusting data for non-parametric index as needed [57]. To investigate the role of symptom severity (ranging from 1 to 3), we performed a one-way ANOVA with Food Refusal, Limited Variety, and food categories accepted as dependent variables. Before running the analysis, we checked the assumptions and normality of residuals, which occurred with Shapiro–Wilk tests and visual inspection. Results indicated that residuals for Food Refusal deviated from normality, whereas those for Limited Variety did not. Homogeneity of variances via Levene’s test occurred for both scales (*p* > 0.05). The first test did not show significant differences across severity levels; however, post hoc Tukey HSD tests were used to detect substantial pairwise differences [57]. Subsequently, we compared both groups using a T-test for the standardized questionnaires, adjusting the analysis to account for the difference between mothers’ and fathers’ responses. Pearson and Spearman correlation coefficients were calculated to examine the relationships between the subscales and the BMI, age classes, percentage of food categories assumed, vomiting, diarrhea, constipation, and reflux. Finally, to identify distinct feeding profiles within each group, a k-means cluster analysis was performed using standardized scores from key variables (k = 3), which were determined based on variance explained (76.7%). To sum up, the analytical strategy examines the complex interaction between food selectivity, sensory processing, gastrointestinal symptoms, dietary habits, BMI, and caregiver feeding practices, avoiding a merely linear model approach [58].

## 3. Results

The defined sample includes 249 records of children with ASD vs. 250 TDC (157/188 mothers), divided into subsamples as shown in Table 1. The age distribution of parents in the entire sample was as follows: 54% (40–49 years), 36% (30–39 years), 9% (50–60 years), and 1% (<30 years). A two-sample proportion test indicated that there is a non-significant, minimal difference between the percentage of ASD (31%) and TDC (31%) of the two groups concerning the initial sample, Z = 0.054, *p* = 0.957.

### 3.1. Clinical Treatment, BMI, GIS, and Symptom Severity

Concerning clinical interventions, the most widely adopted (42%) was Applied Behavior Analysis (ABA) for 32 h a month, along with speech therapy, psychomotor therapy, and parent training (34%). In children under 6 years of age, the prevalence of overweight/obesity was slightly higher in those with ASD compared to TDC. In the 6–8-year age group, children with ASD showed a clear predominance of normal weight, whereas in TDC cases, underweight and overweight were more frequent. Among children aged 9–12 years, a higher proportion of obesity emerged in ASD (10.7%) compared to TDC (8.2%). Table 2 shows all results.

In children younger than 6 years, the prevalence of overweight/obesity was similar between groups (28.1% in ASD vs. 27.5% in TDC). In the 6–8-year age group, however, a marked divergence emerged where only 1.5% of children with ASD were classified as overweight/obese, compared to 26.1% among TDC peers. Among 9–12-year-olds, the prevalence of overweight/obesity was again comparable between groups (21.4% in ASD vs. 20.4% in TDC). Overall, these findings indicate that weight status trajectories differ between ASD and TDC primarily in the 6–8 years developmental window, where ASD children show a markedly lower prevalence of overweight and obesity. Comparing data with Italian published curves, in children under 6, the z-BMI mean was higher in ASD (+0.42) than in TDC (+0.31), while in the 6–8 age range, children with ASD had a z-BMI lower (–0.08) than TDC (+0.12). Finally, in the 9–12 range, in children with ASD, there was a mean increment of z-BMI (+0.56) than TDC (+0.37), consistent with significant obesity. The children followed specific diets, including gluten-free (16/3 cases), casein-free (7/3), and reported milk allergies (6/12), eggs (n = 4/6), fruits/legumes (n = 3/4), and grains (0/10). Finally, seventeen TDC solely reported celiac disease.

Regarding GIS, the majority of children reported never experiencing Vomiting (>83%) since no significant differences were observed between groups in <6 years (χ^2^ (3) = 1.57, *p* = 0.665) or 6–8 years (χ^2^ (6) = 8.11, *p* = 0.230). In the 9–12 age group, a trend was detected, with ASD children reporting often vomiting more frequently than TDC (3.2% vs. 0.0%), but this did not reach significance (χ^2^ (2) = 5.18, *p* = 0.075). In comparison, Diarrhea in the <6 group showed a significant difference (χ^2^ (2) = 6.28, *p* = 0.043), with ASD children reporting diarrhea more frequently (Rarely: 11.8%, Often: 4.5%) than TDC children (Rarely: 7.2%, Often: 0.7%). No significant group differences were observed in the 6–8 (χ^2^ (6) = 5.05, *p* = 0.538) or 9–12 age groups (χ^2^ (2) = 2.94, *p* = 0.230). Regarding Constipation, it was the most prevalent gastrointestinal issue and showed consistent group differences across all age classes. In children <6 years, constipation was significantly higher in ASD (χ^2^ (3) = 28.07, *p* < 0.001), with 22.7% reporting Rarely, 10.9% Sometimes, and 14.5% Often, compared to TDC (16.2%, 4.5%, and 1.3%, respectively). The same pattern held for 6–8 years (χ^2^ (6) = 27.33, *p* < 0.001), where 27.1% of ASD children reported Sometimes or Often constipation versus 8.0% of TDC. In 9–12 years, constipation also remained significantly higher in ASD (χ^2^ (3) = 11.40, *p* = 0.010), with 19.4% reporting Sometimes/Often compared to 6.4% of TDC. Finally, Reflux did not differ significantly between groups in the <6 (χ^2^ (3) = 4.43, *p* = 0.218) or 6–8 (χ^2^ (6) = 3.63, *p* = 0.727) age groups. However, a non-significant trend occurred in the 9–12 years class (χ^2^ (1) = 3.23, *p* = 0.072), with ASD children more often reporting reflux than TDC (Rarely: 8.2% vs. 1.6%).

Lastly, regarding the association between autism severity and food selectivity, the ASD sample was divided into three levels, which did not respect the assumption of proportion: Level 1 (n = 43), Level 2 (n = 135), and Level 3 (n = 53); also, some parents did not report the related scores. A one-way ANOVA between symptom severity levels and Food Refusal (f-ratio = 0.41; *p*. = 0.66), Limited Variety (f-ratio = 0.05; *p*. = 0.95), and Food Categories assumed (f-ratio = 0.05; *p*. = 0.95) did not show a significant association. On the other hand, post hoc Tukey HSD for all scales examined displayed an essential difference between the 1 and 3 levels of severity compared to other comparisons, but this difference was not significant (Q = 0.63; *p* = 0.89), while two high-correlated items such as “My child prefers crunchy foods (e.g., snacks, crackers)” and “My child only likes sweet foods”, showed a significant outcome (f-ratio = 4.27; *p*. = 0.015) in Pairwise Comparisons (level 1/3 = 0.59; Q = 3.61; *p* = 0.030; level 2/3 = 0.58; Q = 3.54; *p* = 0.034).

### 3.2. Differences in Food Selectivity

Comparative analyses revealed significant group differences across all three feeding behavior scales—Food Refusal, Limited Food Variety, and Autism Characteristics—across educational levels (Table 3). Additionally, comparisons between maternal and paternal reports did not yield any statistically significant differences across the three scales of BAMBI. In addition, we examined the within-group comparisons for each scale separately within the ASD and TDC groups to examine whether school level influenced the three scales. Still, no significant differences emerged in either group.

Likewise, we studied via chi-square tests the consumption of bread, fruit, vegetables, dairy, or meat/fish through age classes and groups, where parents’ responses on their child were binary as “eat” and “do not eat”. Results indicate a high proportion of children in both groups consumed bread, where preschoolers with ASD showed slightly higher consumption (98.8%) compared to TDC peers (95.1%). Still, this difference was not significant (*p* > 0.05). At primary school, bread consumption was significantly lower in ASD children (84.9%) compared to TDC (98.7%), χ^2^ = 12.97, *p* < 0.001. In middle school, nearly all children consumed bread (ASD 94.7%, TDC 100.0%), with no significant difference. Vegetable consumption was consistently lower in ASD compared to TDC children across all age classes. In preschool, 65.4% of ASD children consumed vegetables compared to 82.0% of TDC, χ^2^ = 6.12, *p* = 0.013. At primary school, the gap was larger (ASD 56.6% vs. TDC 81.8%), χ^2^ = 19.81, *p* < 0.001. Middle school children showed a similar pattern (ASD 68.4% vs. TDC 86.4%), χ^2^ = 5.11, *p* = 0.024, while fruit consumption also differed by group, with ASD children reporting lower intake. In preschool, 60.5% of ASD vs. 72.1% of TDC consumed fruit, though this difference was not significant (*p* = 0.11). At primary school, the difference was substantial (ASD 54.7% vs. TDC 81.8%), χ^2^ = 23.41, *p* < 0.001. In middle school, ASD consumption remained lower (68.4%) compared to TDC (79.7%), but the difference did not reach statistical significance (*p* = 0.13). In addition, children with ASD showed systematically lower dairy consumption. In preschool, 72.8% of ASD consumed dairy vs. 91.8% of TDC, χ^2^ = 12.56, *p* < 0.001. At primary school, similar differences were observed (ASD 75.5% vs. TDC 93.5%), χ^2^ = 13.31, *p* < 0.001. In middle school, consumption remained lower among ASD (73.7%) compared to TDC (91.5%), χ^2^ = 9.19, *p* = 0.002. A similar pattern was evident for meat/fish consumption. Preschoolers with ASD reported lower intake (86.4%) compared to TDC (93.4%), but the difference was not significant (*p* = 0.12). At primary school, differences were significant (ASD 75.5% vs. TDC 94.8%), χ^2^ = 17.59, *p* < 0.001. In middle school, the pattern persisted (ASD 82.5% vs. TDC 91.5%), though the difference was only marginally significant (*p* = 0.07). To sum up, children with ASD consistently reported lower consumption of vegetables, fruits, dairy, and meat/fish compared to TDC peers, with differences most pronounced at the primary school level. Within-group analyses revealed that for ASD children, consumption of vegetables and fruits significantly varied with age, with a marked reduction during primary school and partial recovery by middle school. Bread, dairy, and meat/fish intake remained relatively stable. For TDC children, only vegetable consumption showed a significant age effect, increasing progressively across school levels. Other food categories remained consistently high across all ages.

### 3.3. Differences in Sensory Processing and Parental Feeding Practices

Sensory processing and feeding practice interaction showed that children with ASD scored significantly lower than TDC, indicating greater sensory processing difficulties. Specifically, significant differences emerged in the Tactile (M = 28.47/32.90, t = −13.64, *p* < 0.001), Taste/Smell (M = 14.85/17.81, t = −8.19, *p* < 0.001), Movement (M = 12.91/14.23, t = −6.72, *p* < 0.001), Sensation Seeking (M = 21.93/29.20, t = −9.72, *p* < 0.001), Auditory Filtering (M = 18.74/26.44, t = −12.00, *p* < 0.001), Weakness (M = 25.50/28.88, t = −6.65, *p* < 0.001), and Visual/Auditory (M = 18.47/22.57,t = −5.29, *p* < 0.001). In terms of caregiver feeding practices, children with ASD reported higher levels of the High Control subscale (M = 5.91/4.09, t = 3.78, *p* < 0.001) and Contingency Management (M = 8.02/6.84, t = 2.18, *p* = 0.029). At the same time, no differences were found in the Child-Centered style (t = 0.36, *p* = 0.713), suggesting that while caregivers of children with ASD may employ more directive or structured feeding strategies, their child-oriented practices do not differ markedly.

### 3.4. Correlations

Firstly, the percentage of food categories consumed negatively correlated with Food Refusal and Limited Variety scales in both groups, where Vomiting was positively associated, while other GIS, such as Diarrhea, Constipation, and Reflux, correlated significantly solely in the TDC group. Strong and consistent correlations emerged between sensory processing scales and feeding problems. In the TDC group, all subscales showed significant positive correlations with Food Refusal, particularly Taste/Smell, Tactile, and Auditory Filtering. In the ASD group, Food Refusal was moderately associated with Tactile, Auditory Filtering, and Visual/Auditory, while Limited Variety was most strongly associated with Taste/Smell Sensitivity. In terms of caregiver feeding practices, higher levels of the High Control and Contingency Management were significantly associated with Food Refusal in both groups. At the same time, the Child-centered style showed positive associations with both Food Refusal and Limited Variety (please see Table 4). No significant differences emerged between boys and girls across any dimension in children with ASD while in TDC, boys and girls differed in Limited Variety (χ^2^ = 5.42, *p* = 0.02) and High Control (χ^2^ = 4.42, *p* = 0.036), with girls more frequently classified as above the mean for Limited Variety, and boys above the mean for High Control.

### 3.5. Clustering

The cluster analysis was performed separately for both groups using the following input variables: age, BMI, diet, sensory profiles, gastrointestinal symptoms, mealtime behavior scales, and caregiver feeding practices. The cluster solution identified three distinct profiles: children with ASD involved the first a high-risk profile (9.9% out of the subsample), an intermediate (35.6%), and a protective profile (54.5%). Children with ASD in the high-risk profile showed higher levels of food refusal than other clusters, a restricted diet, and higher gastrointestinal symptoms, along with taste, smell, tactile, and auditory oversensitivity, and higher levels of parental feeding styles. Among TDC, high-risk (2.5%), intermediate (38.2%), and protective (59.3%) profiles were identified, with the high-risk profile exhibiting a pattern similar to the first cluster in children with ASD, albeit with lower levels of food selectivity and a diet that was sufficient, albeit limited. Also, GIS resulted in lower values, except for Reflux. The other two clusters gradually showed improvements in diet variety for both groups, a lower GIS score, except for Constipation, and parental practices were less authoritarian (Table 5 shows the comparison).

## 4. Discussion

This study investigated via survey food selectivity, restricted diets, anthropometric curves, sensory processing, gastrointestinal symptoms, and parental feeding practices, which are critical practical issues in daily life in children with ASD. We compared children with ASD with TDC, matched by gender and age range, offering a comprehensive analysis of the phenomenon and its multidimensional correlates. The findings extend prior research by examining behavioral, sensory, physiological, and parental feeding styles across different age ranges in childhood [59]. Consistent with previous literature, children with ASD exhibited significantly higher rates of food refusal and limited dietary variety compared to TDC regarding the first research question [4,5,6,18], showing stability through age classes. In addition, both parents responded similarly to the items, likely because mealtimes occur more frequently during the day, when parents commonly share concerns about feeding and nutritional issues of their child. These results are in line with previous studies, indicating a long-term persistence of food selectivity in children with ASD [60]. The factors that moderate this difference between groups have been explored in further analyses.

In fact, regarding sensory sensitivities and feeding behavior, the results confirmed greater sensory difficulties in children with ASD compared with TDC. Significant group differences occurred across all SSP subscales—particularly tactile, taste/smell, and auditory sensitivity, confirming the link between sensory processing issues and food selectivity in ASD [61]. These findings align with prior studies indicating that sensory over-responsivity is a central contributor to food selectivity [15,28,36,45]. Specifically, the positive correlations between tactile and taste sensitivities and BAMBI scales in both groups highlight how sensory-based aversions may drive mealtime refusal and reduce food variety. This suggests that sensory processing patterns may serve as a shared mechanism for selective eating across neurodevelopmental profiles, though more pronounced in ASD.

Likewise, concerning gastrointestinal issues, the study showed that all symptoms might result in feeding difficulties, even in the TDC group. This finding aligns with prior literature suggesting a bidirectional relationship between food selectivity and GIS [23,24,25]. Gastrointestinal problems are highly prevalent in individuals with ASD, with estimates varying across studies but consistently exceeding those observed in the general population [62]. The most reported symptom is constipation, which is often chronic and sometimes leads to encopresis (fecal incontinence), alternating with diarrhea. Other abdominal discomforts are generalized stomachaches, bloating, gas, or other signs of distress. Such symptoms can be particularly challenging to identify in non-verbal individuals with ASD, who may express them through behavioral changes such as increased irritability, self-biting, mouthing objects, aggression, or repetitive behaviors [63]. In fact, comparisons showed that children with ASD often reported diarrhea and constipation more frequently than TDC, and gastrointestinal issues were prevalent in high-risk clusters of food selectivity in both groups of children. The reasons for the greater prevalence of GIS in ASD are still not fully understood, but constipation can be induced by a diet poorer in fiber and legumes throughout childhood. Correspondingly, imbalances in gut microbiota are observed in ASD and linked to gastrointestinal issues [64], and heightened sensory perception can make certain foods intolerable, leading to restricted diets that may exacerbate them, causing children to avoid foods with specific colors, smells, temperatures, and tastes (preferring crunchy and sweet foods like bread, crackers, and chips).

On the other hand, it is still unclear how a limited diet affects anthropometric curves and nutritional intake of children. Although BMI was not significantly related to food refusal or limited variety, children with higher feeding selectivity consumed a narrower range of food categories, which may increase the risk of nutritional deficiencies, especially in fiber, calcium, and vitamins, as noted in prior meta-analyses [16,22], even if we did not gather nutritional intake. Concerning the categories of food accepted by children, those with ASD ate considerably less vegetables, fruit, meat, fish, and dairy than TDC, which increased vegetable intake through age classes. The current survey reports a scarce vegetable intake and the presence of underweight and obesity in children with ASD, in line with the literature, although prior studies, including even adolescents [17,18]. Moreover, specific age groups exhibited BMI issues in both groups, likely influenced by changes in feeding routines, such as behaviors modeled by peers in school canteens. In addition, the severity of autism might moderate these results since we discovered associations between low-functioning children and consumption of junk food, but served a larger subsample since our subsamples did not respect psychometric assumptions.

Importantly, the researchers should not consider the study of these dimensions as bidirectional only, but as a multidimensional phenomenon in terms of change if treatment in one or more dimensions is established at a particular time. For example, this study examined the role of parental feeding styles using the CFSQ. Caregivers of children with ASD were significantly more likely to employ high-control and contingency management strategies, consistent with prior findings that parents often adopt more directive styles in response to perceived feeding challenges [33,55]. Significantly, both feeding styles were positively correlated with higher food refusal and lower dietary variety in children, regardless of diagnosis, suggesting that such approaches may inadvertently reinforce selective behaviors. Although parents of children with ASD address high stress pre- and during mealtime, they may not require a feeding intervention for the anxiety of seeing a regression in the child, as well as to accommodate the child’s daily requests. These results support models that emphasize the importance of reciprocal parent–child dynamics in maintaining feeding issues [30,35]. Moreover, our findings underscore the need for intervention models that integrate behavioral, physical, and sensory strategies with parental coaching to promote flexible eating habits and mitigate mealtime conflict (to respond to research question 2).

One of the most novel aspects of this study was the identification of distinct subgroups through cluster analysis (research question 3). In both ASD and TDC samples, an exhaustive three-cluster solution emerged, delineating profiles ranging from “typical” to “high-risk”. In the ASD group, severe food selectivity, significant sensory deficits, and frequent GIS marked the high-risk cluster. These children consumed poor food categories and scored one SD below the normative mean in taste and tactile sensitivity. These findings align with those from previous clustering studies, underscoring the heterogeneity within the ASD population and the utility of multidimensional profiling for clinical triage [26,28]. In the TDC group, a smaller high-risk cluster exhibited a similarly maladaptive profile but with lower symptoms, suggesting that while food selectivity is more prevalent in ASD, its moderate forms may also exist in neurotypical children, often overlooked in clinical practice. These insights indicate that feeding issues beyond diagnostic categories should integrate sensory, behavioral, and gastrointestinal domains to better tailor interventions.

### Limitations of the Study and Future Direction of Research

Several limitations of our study should be noted. Firstly, the study relied on parent-reported measures, which may be subject to social desirability or rater bias. Although maternal and paternal scores were compared and found to be consistent, prior research suggests that maternal burden may influence perceptions of feeding behaviors [40]. Second, the cross-sectional design limits the ability to make causal inferences. Longitudinal studies are needed to track the stability of feeding profiles over time and their response to interventions [65]. Third, the lack of adolescents or young adults may restrict generalizability to older age groups. Lastly, the analysis focused on three core BAMBI subscales, excluding the Autism Characteristics factor due to its low internal consistency, which suggests the need for refined measurement tools in this domain [43,44]. The results obtained from current indirect measures may differ from direct observations, particularly for diet, anthropometric curves, and gastrointestinal issues.

As a possible future direction of research, our study might foster early identification and highly personalized and multidisciplinary interventions for high-risk feeding profiles, which may mitigate long-term nutritional, behavioral, and psychological outcomes in both ASD and TDC populations. Moreover, future studies might prioritize multimodal assessments (e.g., observational, physiological, microbiome analysis) and evaluate integrative interventions that combine dietary, sensory, and behavioral components [66]. Investigations into the role of caregiver stress and cultural feeding norms could also enhance understanding of cross-contextual influences on child eating behaviors. Conversely, strengths may include addressing a timely and clinically significant topic in autism research, such as feeding difficulties and their multidimensional correlates, which can be compared across several subsamples, including those with and without neuropsychiatric issues. Furthermore, a comprehensive methodology was employed, utilizing a large sample with matched and paired controls, using standardized tools and qualitative reports (consideration of both parent genders in reporting). Additionally, an analytical rigor is employed, utilizing multiple statistical approaches (t-tests, ANOVA, correlations, and cluster analysis), including subgroup analyses by age and severity. Cluster analysis yields novel insights into feeding profiles and their interpretation, along with a synthesis of the literature that presents practical implications for intervention.

## 5. Conclusions

This study highlights the multidimensional nature of feeding problems in children with ASD, showing that food selectivity is closely linked to sensory hypersensitivity, gastrointestinal symptoms, and parental feeding practices. A high-risk cluster characterized by behavioral rigidity, sensory over-responsivity, and somatic distress was identified, although less pronounced profiles were also observed among typically developing children. Notably, no associations emerged between BMI and feeding difficulties, restricted food categories, or gastrointestinal problems, underscoring the need to look beyond anthropometric measures when evaluating nutritional risk. These findings provide novel insights with direct implications for clinical practice. Interventions targeting feeding difficulties in ASD should adopt a multidisciplinary framework that integrates behavioral, nutritional, sensory, and parental components. Future research should extend these results by examining longitudinal trajectories, exploring biological correlates such as gut microbiota, and testing the effectiveness of tailored intervention models for both children with ASD and typically developing peers.

## Figures and Tables

**Table 1:** Defined sampling for analyses.

Samples	Preschool			Primary			Middle		
	M	F	Total	M	F	Total	M	F	Total
ASD	97 *	15	112	60	14	74	46	17	63
TDC	67 *	15	82	69	21	90	51	27	78
N.	164	30	194	129	45	164	97	44	142

Note: Extracted proportions of both groups were similar. * There was only a significantly slight difference in gender between preschooler males with ASD (50%) and males with TDC (35%), Z = 3.08, *p* = 0.002.

**Table 2:** Differences between ASD and TDC on BMI by age classes.

Sample	Age Class	Underweight	Normal	Overweight	Obese
	Preschool	7.1%	64.8%	22.3%	5.8%
ASD	Primary	0.0%	97.9%	0.6%	1.5%
	Middle	0.0%	67.9%	21.4%	10.7%
	Preschool	5.8%	66.7%	18.8%	8.7%
TDC	Primary	6.2%	67.7%	20.0%	6.2%
	Middle	4.1%	67.3%	20.4%	8.2%
**Sample**	**Age Class**	**Mean z-BMI**		**Standard Dev.**	
	Preschool	+0.42		1.15	
ASD	Primary	–0.08		0.94	
	Middle	+0.56		1.21	
	Preschool	+0.31		1.05	
TDC	Primary	+0.12		1.08	
	Middle	+0.37		1.14	

Note: A chi-square was conducted on dichotomized BMI categories (Overweight/Obese vs. Not Overweight/Obese), stratified by Age Class and Sample. Analysis revealed a statistically significant overall difference across groups (χ^2^ (5) = 24.8, *p* < 0.001, Cramér’s V = 0.16), suggesting that distribution of weight status varied by age and diagnostic condition.

**Table 3:** Differences between ASD and TDC on BAMBI.

Scales	School	ASD	TDC	*t* test	*p*
	Preschool	8.59 (3.74)	6.50 (2.37)	4.024	0.000 ***
Refusal	Primary	8.09 (2.96)	5.73 (1.62)	6.834	0.000 ***
	Middle	7.07 (2.60)	5.48 (1.80)	3.333	0.000 ***
	Preschool	19.8 (7.71)	15.8 (4.90)	3.541	0.000 ***
Limited Variety	Primary	19.3 (5.16)	19.1 (5.20)	5.459	0.000 ***
	Middle	20.6 (5.99)	16.7 (6.08)	2.831	0.004 **
	Preschool	9.59 (3.23)	7.78 (2.54)	3.527	0.000 ***
Autism Characteristics	Primary	9.77 (2.59)	7.65 (2.54)	5.999	0.000 ***
	Middle	10.1 (3.30)	7.45 (2.68)	4.013	0.000 ***
		**Mothers/Fathers**		***t* test**	***p***
Refusal	Sample	7.7/7.34	5.7/5.71	0.7/0.8	0.30
Limited Variety	Sample	19.8/19.9	16.5/16.3	0.05/0.7	0.50
Autism Characteristics	Sample	9.7/9.8	7.6/7.75	0.2/0.9	0.35

Note: alpha values = ** < 0.01; *** = <0.001.

**Table 4:** Correlations.

Measures	Scales	Refusal		Limited Variety	
		**ASD**	**TDC**	**ASD**	**TDC**
	Age	−0.19 **	−0.21 **	−0.04	0.07
	Body Mass Index	−0.098	−0.087	−0.080	−0.018
	Food Categories (%)	−0.252 **	−0.348 **	−0.319 **	−0.419 **
SURVEY	Vomiting	0.264 **	0.274 **	0.070	0.235 **
	Diarrhea	0.096	0.153 *	0.121	0.158 *
	Reflux	0.129	0.131 *	0.108	0.186 **
	Constipation	0.143	0.213 **	0.172	0.164 **
	Tactile Sensitivity	0.442 **	0.484 **	0.183	0.365 **
	Taste/Smell	0.179	0.606 **	0.494 **	0.595 **
SSP	Movement	0.189 *	0.232 **	0.092	0.194 **
	Seek Sensation	0.137	0.437 **	0.191 *	0.293 **
	Auditory Filtering	0.268 **	0.431 **	0.292 **	0.284 **
	Low Energy/Weak	0.161	0.277 **	0.015	0.161 *
	Visual/Auditory	0.207 *	0.470 **	0.164	0.268 **
	High control	0.486 **	0.644 **	0.177	0.425 *
CFSQ	Contingency Management	0.434 **	0.545 ***	0.269 **	0.487 **
	Child Centered	0.242 *	0.432 **	0.353 **	0.321 **

Note: alpha values = * < 0.05; ** < 0.01; *** = <0.001. Additionally, Vomiting in ASD sample correlated with Reflux (0.522 **), grains (0.476 **), fruit (0.402 **), and diet (−0.411 **), while in the TDC sample, it correlated with Diarrhea (0.451 **) and Reflux (0.438 **).

**Table 5:** Comparison of cluster solutions between ASD and TDC.

	ASD			TDC		
Variables	High-Risk	Intermediate	Protective	High-Risk	Intermediate	Protective
Age (years)	7.77 (2.75)	6.37 (2.65)	8.09 (2.92)	7.96 (5.24)	6.91 (2.69)	8.09 (3.06)
BMI (kg/m^2^)	18.03 (4.37)	16.49 (4.87)	17.93 (5.26)	17.45 (3.74)	15.89 (3.53)	17.82 (4.48)
Food Categories	27% (57.4)	50% (50.8)	75% (38.8)	60% (36.9)	61% (63.1)	88% (31.4)
Food Refusal	12.14 (3.32)	10.2 (3.02)	6.05 (1.46)	9.0 (3.61)	6.88 (1.94)	5.08 (0.83)
Limited Variety	25.14 (4.83)	23.73 (5.87)	16.82 (4.63)	24.91 (5.38)	19.61 (4.94)	14.29 (4.0)
Tactile *	26.9 (4.5)	26.79 (4.75)	30.1 (3.57)	29.09 (5.61)	32.1 (3.32)	34.04 (1.38)
Taste/Smell *	8.86 (3.84)	13.49 (4.31)	17.18 (3.76)	14.45 (5.18)	15.74 (3.67)	19.38 (1.37)
Auditory Filtering *	16.24 (5.02)	17.27 (4.43)	20.66 (5.83)	25.45 (4.23)	24.79 (4.19)	27.72 (2.81)
Vomiting	1.29 (0.78)	0.0 (0.0)	0.03 (0.18)	0.91 (0.7)	0.09 (0.28)	0.02 (0.15)
Diarrhea	0.76 (0.77)	0.19 (0.51)	0.1 (0.32)	0.55 (0.82)	0.15 (0.37)	0.05 (0.22)
Constipation	1.29 (1.15)	0.88 (1.03)	0.73 (1.13)	0.91 (0.7)	0.44 (0.71)	0.22 (0.57)
Reflux	1.0 (1.26)	0.12 (0.52)	0.13 (0.52)	2.73 (1.27)	0.02 (0.13)	0.04 (0.27)
High Control	2.84 (0.89)	2.61 (0.85)	1.44 (0.5)	2.24 (1.08)	1.71 (0.6)	1.12 (0.24)
Conting. Management	2.44 (0.84)	2.52 (0.93)	1.58 (0.61)	2.32 (1.13)	2.28 (0.72)	1.38 (0.45)
Child-Centered	3.52 (0.93)	3.18 (0.5)	2.56 (0.72)	3.17 (1.14)	3.19 (0.65)	2.34 (0.72)

Note: * Lower scores indicate higher sensory issues. Scores show mean ± SD comparing data solely in rows rather than columns.

## Data Availability

The database is available upon reasonable request to the corresponding author. The datasets generated and analyzed during the current study are not publicly available, as parental consent was required for data collection, analysis, and publication of results. Data cannot be shared, even in anonymized form, without further authorization.
